# Surgical tactics in treatment of traumatic raptures of the diaphragm: our experience

**DOI:** 10.1186/1749-8090-8-S1-P38

**Published:** 2013-09-11

**Authors:** M Mkrtchyan, H Sarkavagyan, T Khachatryan, A Khanoyan, A Manukyan, A Sardaryan, H Kikoyan

**Affiliations:** 1 Department of Thoracic Surgery, St. Grigor Lusavorich MC, Yerevan, Armenia

## Background

The aim of this retrospective study was to analyze our experience with operative approach of traumatic rapture of diaphragm (TRD).

## Methods

38 patients with TRD (34 men and 4 women ranging from 13 to 70 years) were treated in 1993-2013. 29 patients (76%) had a left TRD and 9 patients (24%) - right TRD. Multiple-associated injures were observed in 31 patients (82%), and isolated TRD - in 7 patients (18%). Causes of trauma included vehicle crash for 33 patients and fall from height for 5.

## Results

TRD was diagnosed preoperatively in 32 patients (84%) by contrast X-Ray of gastrointestinal tract, abdominal ultrasound, and CT scan of the chest and abdomen. In 6 (16%) patients TRD was diagnosed during surgery. We did not use pleural centesis to avoid injury of abdominal organs. 27 patients (71%) underwent surgery upon 1 month of trauma episode, and 13 (34%) – after 1 month to 13 years. Right lateral thoracotomy on the 6th interspace was performed in 9 (24%) patients with right TRD. In case of large raptures the diaphragm was repaired by simple interrupted suture to chest wall on 1-2 interspaces above anatomical juncture-line which allowed repairing the diaphragm out of high tension. 11 patients (29%) with old left TRD underwent left lateral thoracotomy on the 6th interspace. In both left and right TRDs the large diaphragmatic defects were repaired by polypropylene mesh. 18 (47%) patients with acute left TRD were treated by left lateral thoracotomy accompanied by upper-medial laparotomy (11 cases) and laparoscopy (7 cases) for better examination of abdomen and restoring lesions. We observed 3 deaths (8 %) – 2 from severe craniocerebral trauma and 1 from pulmonary thromboemboli.

## Conclusions

Our experience showed superiority of repairing of diaphragm on 1-2 interspaces above anatomical juncture-line in right TRD to avoid hypertension of sutures and accompanying thoracotomy with laparoscopy in left TRD as a rational surgical approach.

